# On Quasi-Cyclic Codes of Index 3

**DOI:** 10.3390/e27111096

**Published:** 2025-10-23

**Authors:** Kanat Abdukhalikov, Rasha M. Shat

**Affiliations:** Department of Mathematical Sciences, UAE University, Al Ain P.O. Box 15551, United Arab Emirates

**Keywords:** quasi-cyclic codes, dual codes, one-generator codes, 94B05, 94B15, 94B60

## Abstract

Quasi-cyclic codes of index 3 over finite fields are studied. We give a classification of such codes. Their duals with respect to the Euclidean and Hermitian inner products are investigated. We give a characterization of self-orthogonal and dual-containing codes. A quasi-cyclic code of index 3 is generated by at most three elements. We describe conditions when such a code (or its dual) is generated by one element.

## 1. Introduction

Quasi-cyclic (QC) codes are an important type of linear codes, which is a generalization of cyclic codes. There are families of QC codes that are asymptotically good [1,2]. Many good codes have been constructed with a better minimum distance than any linear code of the same length and dimension previously constructed. Ling and Solé explored the algebraic structure of QC codes in-depth in a series of articles [3,4,5,6]. In [7,8], the algebraic structure of quasi-cyclic codes was discussed. A class of 1-generator quasi-cyclic codes and their properties were studied in [9]. Some classes of quasi-cyclic codes of index 2 were considered in [10,11,12]. Quasi-cyclic codes of index 2 were fully investigated in [13,14]. Lally [15] presented lower bounds for quasi-cyclic codes. In [16,17], distance bounds for quasi-twisted codes were studied. Aydin et al. [18] studied the structure of 1-generator quasi-twisted codes and constructed some new linear codes. In [19], QC perfect codes in Doob graphs and special partitions of Galois rings were considered.

Dastbasteh and Shivji [20] considered additive cyclic codes over $F_{p^{2}}$ that can be considered as an interpretation of QC codes over $F_{p}$. They computed the symplectic dual of additive codes by decomposing them into components.

Lally [15] presented lower bounds for the minimum distance of quasi-cyclic codes. Semenov and Trifonov [21] introduced a spectral method for quasi-cyclic codes and obtained a BCH-like bound. This has led to several further works on distance bounds, known as spectral bounds, for quasi-cyclic and quasi-twisted codes, e.g., [16,17,22,23,24]. Yet another approach, using the concatenated structure of quasi-cyclic codes, also yields a different type of distance lower bound for quasi-cyclic codes, e.g., [25].

Hermitian duals of one-generator quasi-twisted codes of index 3 were considered in [26]. Shi et al. [27] considered additive cyclic codes over $F_{q^{3}}$ under the condition gcd(m,q)=1.

In this paper we consider QC codes of index 3 in full generality. Unlike other papers, where the condition gcd(m,q)=1 is assumed, we do not have restrictions on the characteristic of the ground field, except for the case of one-generator codes (Theorems 3 and 5). More importantly, this approach is simpler and more powerful. The paper is organized as follows. We recall first in Section 2 definitions and notation concerning quasi-cyclic codes. In Section 3 we give a classification of QC codes of index 3 over finite fields. A QC code of index 3 is generated by at most three elements. We give necessary and sufficient conditions for a QC code to be generated by one element. For this, we do not need extra assumptions such as decomposition of codes into component subcodes, and we only use generators of codes. We describe duals of QC codes with respect to the Euclidean and Hermitian inner products in Section 4 and Section 5, respectively. We study duals of QC codes in a systematic way involving the notion of adjoint maps. Moreover, we describe self-orthogonal and dual-containing QC codes. Finally, in Section 6 we summarize our results.

## 2. Preliminaries

Let $F=F_{q}$ be a finite field of *q* elements. A linear code *C* of length *n* is a subspace of the vector space $F^{n}$. The elements of *C* are called codewords.

Let *T* be the standard cyclic shift operator on $F^{n}$. A code is said to be *quasi-cyclic* (QC) of index *ℓ* if it is invariant under $T^{\ell }$. We can assume that *ℓ* divides *n*. If ℓ=1 then the QC code is a cyclic code.

Let $R=F\left[x\right]/\langle x^{m}-1\rangle$. We recall that cyclic codes of length *m* over *F* can be considered as ideals of *R*.

Let n=mℓ and let *C* be a linear QC code of length mℓ and index *ℓ* over *R*. Let

$$c=(c_{0,0},c_{0,1},\ldots ,c_{0,\ell -1},c_{1,0},c_{1,1},\ldots c_{1,\ell -1},\ldots ,c_{m-1,0},c_{m-1,1},\ldots ,c_{m-1,\ell -1})$$

denote a codeword in *C*. Define a map $\varphi :F^{m\ell }\to R^{\ell }$ by

$$\varphi \left(c\right)=\left(c_{0}\left(x\right),c_{1}\left(x\right),\ldots ,c_{\ell -1}\left(x\right)\right)\in R^{\ell },$$

where

$$c_{j}\left(x\right)=c_{0,j}+c_{1,j}x+c_{2,j}x^{2}+\cdots +c_{m-1,j}x^{m-1}\in R.$$

The following Lemma is well-known.

**Lemma** **1** ([3,8])**.** *The map φ induces a one-to-one correspondence between linear QC codes over F of index ℓ and length mℓ, and linear codes over R of length ℓ.*

## 3. Quasi-Cyclic Codes of Index 3

The next Theorem gives a description of QC codes over *F* of index 3.

**Theorem** **2.** *(1) Let C be a QC code of length 3m and index 3. Then C is generated by three elements $g_{1}=\left(g_{11}\left(x\right),g_{12}\left(x\right),g_{13}\left(x\right)\right)$, $g_{2}=\left(0,g_{22}\left(x\right),g_{23}\left(x\right)\right)$ and $g_{3}=\left(0,0,g_{33}\left(x\right)\right)$ such that they satisfy the following conditions:*

(∗) $$\begin{array}{c} g_{11}\left(x\right)|\left(x^{m}-1\right),\;g_{22}\left(x\right)|\left(x^{m}-1\right)\;\mathrm{and}\;g_{33}\left(x\right)|\left(x^{m}-1\right),\\ \deg g_{12}\left(x\right)<\deg g_{22}\left(x\right),\;\deg g_{13}\left(x\right)<\deg g_{33}\left(x\right),\;\deg g_{23}\left(x\right)<\deg g_{33}\left(x\right),\\ g_{11}\left(x\right)g_{22}\left(x\right)|\left(x^{m}-1\right)g_{12}\left(x\right),\;g_{22}\left(x\right)g_{33}\left(x\right)|\left(x^{m}-1\right)g_{23}\left(x\right),\\ g_{11}\left(x\right)g_{22}\left(x\right)g_{33}\left(x\right)|\left(x^{m}-1\right)\left(g_{12}\left(x\right)g_{23}\left(x\right)-g_{13}\left(x\right)g_{22}\left(x\right)\right). \end{array}$$

*Moreover, in this case $\dim C=3m-\deg g_{11}\left(x\right)-\deg g_{22}\left(x\right)-\deg g_{33}\left(x\right).$*

*(2) Let code C be generated by elements $g_{1}=\left(g_{11}\left(x\right),g_{12}\left(x\right),g_{13}\left(x\right)\right)$, $g_{2}=\left(0,g_{22}\left(x\right),g_{23}\left(x\right)\right)$ and $g_{3}=\left(0,0,g_{33}\left(x\right)\right)$, and let $C^{\prime }$ be generated by elements $g_{1}^{\prime }=\left(g_{11}^{\prime }\left(x\right),g_{12}^{\prime }\left(x\right),g_{13}^{\prime }\left(x\right)\right)$, $g_{2}^{\prime }=\left(0,g_{22}^{\prime }\left(x\right),g_{23}^{\prime }\left(x\right)\right)$ and $g_{3}^{\prime }=\left(0,0,g_{33}^{\prime }\left(x\right)\right)$, both satisfying Conditions (∗). Let $g_{11}\left(x\right)$, $g_{22}\left(x\right)$, $g_{33}\left(x\right)$, $g_{11}^{\prime }\left(x\right)$, $g_{22}^{\prime }\left(x\right)$$g_{33}^{\prime }\left(x\right)$ be monic polynomials. Then $C=C^{\prime }$ if and only if $g_{11}\left(x\right)=g_{11}^{\prime }\left(x\right)$, $g_{22}\left(x\right)=g_{22}^{\prime }\left(x\right)$, $g_{33}\left(x\right)=g_{33}^{\prime }\left(x\right)$, $g_{12}\left(x\right)=g_{12}^{\prime }\left(x\right)$, $g_{12}\left(x\right)=g_{12}^{\prime }\left(x\right)$, $g_{23}\left(x\right)=g_{23}^{\prime }\left(x\right)$.*

**Proof.**  (1) By [8] (Theorem 2.1) we can assume that *C* is generated by three elements $g_{1}=\left(g_{11}\left(x\right),g_{12}\left(x\right),g_{13}\left(x\right)\right)$, $g_{2}=\left(0,g_{22}\left(x\right),g_{23}\left(x\right)\right)$ and $g_{3}=\left(0,0,g_{33}\left(x\right)\right)$, satisfying the conditions $g_{11}\left(x\right)|\left(x^{m}-1\right),\;g_{22}\left(x\right)|\left(x^{m}-1\right)\;\mathrm{and}\;g_{33}\left(x\right)|\left(x^{m}-1\right)$, $\deg g_{12}\left(x\right)<\deg g_{22}\left(x\right),\;\deg g_{13}\left(x\right)<\deg g_{33}\left(x\right),\;\deg g_{23}\left(x\right)<\deg g_{33}\left(x\right)$, and $\dim C=3m-\deg g_{11}\left(x\right)-\deg g_{22}\left(x\right)-\deg g_{33}\left(x\right)$. Existence of such generators is equivalent [8] (Theorems 2.1 and 2.2) to the existence of a 3×3 polynomial matrix $\left(a_{ij}\right)\in {\mathrm{Mat}}_{3}\left(F\left[x\right]\right)$ such that

$$\;\left(\begin{array}{ccc} a_{11}\left(x\right) & a_{12}\left(x\right) & a_{13}\left(x\right)\\ 0 & a_{22}\left(x\right) & a_{23}\left(x\right)\\ 0 & 0 & a_{33}\left(x\right) \end{array}\right)\left(\begin{array}{ccc} g_{11}\left(x\right) & g_{12}\left(x\right) & g_{13}\left(x\right)\\ 0 & g_{22}\left(x\right) & g_{23}\left(x\right)\\ 0 & 0 & g_{33}\left(x\right) \end{array}\right)=\left(\begin{array}{ccc} x^{m}-1 & 0 & 0\\ 0 & x^{m}-1 & 0\\ 0 & 0 & x^{m}-1 \end{array}\right).$$

Then

$$a_{11}\left(x\right)=\frac{x^{m}-1}{g_{11}\left(x\right)},\;a_{22}\left(x\right)=\frac{x^{m}-1}{g_{22}\left(x\right)},\;a_{33}\left(x\right)=\frac{x^{m}-1}{g_{33}\left(x\right)},$$

$$a_{11}\left(x\right)g_{12}\left(x\right)+a_{12}\left(x\right)g_{22}\left(x\right)=0,$$

$$a_{22}\left(x\right)g_{23}\left(x\right)+a_{23}\left(x\right)g_{33}\left(x\right)=0,$$

$$a_{11}\left(x\right)g_{13}\left(x\right)+a_{12}\left(x\right)g_{23}\left(x\right)+a_{13}\left(x\right)g_{33}\left(x\right)=0.$$

Therefore,

$$\begin{array}{ccc} a_{12}\left(x\right) & = & -\frac{\left(x^{m}-1\right)g_{12}\left(x\right)}{g_{11}\left(x\right)g_{22}\left(x\right)},\\ a_{23}\left(x\right) & = & -\frac{\left(x^{m}-1\right)g_{23}\left(x\right)}{g_{22}\left(x\right)g_{33}\left(x\right)},\\ a_{13}\left(x\right) & = & \frac{\left(x^{m}-1\right)\left(g_{12}\left(x\right)g_{23}\left(x\right)-g_{13}\left(x\right)g_{22}\left(x\right)\right)}{g_{11}\left(x\right)g_{22}\left(x\right)g_{33}\left(x\right)}. \end{array}$$

Since $a_{ij}\left(x\right)\in F\left[x\right]$, the divisibility conditions from (∗) follow.

(2) Consider the projection mappings $P_{1}:C\to R$, $P_{1}\left(a_{1}\left(x\right),a_{2}\left(x\right),a_{3}\left(x\right)\right)=a_{1}\left(x\right)$, and $P_{12}:C\to R^{2}$, $P_{12}\left(a_{1}\left(x\right),a_{2}\left(x\right),a_{3}\left(x\right)\right)=\left(a_{1}\left(x\right),a_{2}\left(x\right)\right)$. Then

$$\begin{array}{ccc} \dim C & = & \dim P_{1}\left(C\right)+\dim\ker P_{1}\\ & = & \dim P_{1}\left(C\right)+\dim P_{12}\left(\ker P_{1}\right)+\dim\ker P_{12}\\ & \geq & \dim\langle g_{11}\left(x\right)\rangle +\dim\langle \left(0,g_{22}\left(x\right)\right)\rangle +\dim\langle \left(0,0,g_{33}\left(x\right)\right)\rangle \\ & = & \left(m-\deg g_{11}\left(x\right)\right)+\left(m-\deg g_{22}\left(x\right)\right)+\left(m-\deg g_{33}\left(x\right)\right). \end{array}$$

Since $\dim C=\left(m-\deg g_{11}\left(x\right)\right)+\left(m-\deg g_{22}\left(x\right)\right)+\left(m-\deg g_{33}\left(x\right)\right)$, we have that

$$\begin{array}{ccc} \dim P_{1}\left(C\right) & = & m-\deg g_{11}\left(x\right),\\ \dim P_{12}\left(\ker P_{1}\right) & = & m-\deg g_{22}\left(x\right),\\ \dim\ker P_{12} & = & m-\deg g_{33}\left(x\right). \end{array}$$

Therefore,

(1) $$\begin{array}{ccc} P_{1}\left(C\right) & = & \langle g_{11}\left(x\right)\rangle , \end{array}$$

(2) $$\begin{array}{ccc} P_{12}\left(\ker P_{1}\right) & = & \langle \left(0,g_{22}\left(x\right)\right)\rangle , \end{array}$$

(3) $$\begin{array}{ccc} \ker P_{12} & = & \langle \left(0,0,g_{33}\left(x\right)\right)\rangle . \end{array}$$

Similar statements are true for $C^{\prime }$, where $C=C^{\prime }$. Hence, $g_{11}\left(x\right)=g_{11}^{\prime }\left(x\right)$, $g_{22}\left(x\right)=g_{22}^{\prime }\left(x\right)$, $g_{33}\left(x\right)=g_{33}^{\prime }\left(x\right)$.

Further, if $(0,g_{22}\left(x\right),g_{23}\left(x\right))\in C$ and $(0,g_{22}\left(x\right),g_{23}^{\prime }\left(x\right))\in C$, then $g_{23}\left(x\right)-g_{23}^{\prime }\left(x\right)$ is divisible by $g_{33}\left(x\right)$, thus $g_{23}\left(x\right)=g_{23}^{\prime }\left(x\right)$. Similarly, if $(g_{11}\left(x\right),g_{12}\left(x\right),g_{13}\left(x\right))\in C$ and $(g_{11}\left(x\right),g_{12}^{\prime }\left(x\right),g_{13}^{\prime }\left(x\right))\in C$, then $g_{12}\left(x\right)-g_{12}^{\prime }\left(x\right)$ is divisible by $g_{22}\left(x\right)$, thus $g_{12}\left(x\right)=g_{12}^{\prime }\left(x\right)$. Likewise, $g_{13}\left(x\right)=g_{13}^{\prime }\left(x\right)$. □

**Remark** **1.** *If gcd(q,m)=1 then $x^{m}-1$ has no multiple roots, thus the condition $g_{11}\left(x\right)g_{22}\left(x\right)|\left(x^{m}-1\right)g_{12}\left(x\right)$ is equivalent to the condition $\gcd\left(g_{11}\left(x\right),g_{22}\left(x\right)\right)|g_{12}\left(x\right)$, and the condition $g_{22}\left(x\right)g_{33}\left(x\right)|\left(x^{m}-1\right)g_{23}\left(x\right)$ is equivalent to the condition $\gcd\left(g_{22}\left(x\right),g_{33}\left(x\right)\right)|g_{23}\left(x\right)$.*

**Theorem** **3.** *Let gcd(q,m)=1. Let C be a QC code of length 3m and index 3, generated by elements $g_{1}=\left(g_{11}\left(x\right),g_{12}\left(x\right),g_{13}\left(x\right)\right)$, $g_{2}=\left(0,g_{22}\left(x\right),g_{23}\left(x\right)\right)$ and $g_{3}=\left(0,0,g_{33}\left(x\right)\right)$, satisfying Conditions (∗). Then C is generated by one element if and only if $g_{11}\left(x\right)g_{22}\left(x\right)\equiv g_{11}\left(x\right)g_{33}\left(x\right)\equiv g_{22}\left(x\right)g_{33}\left(x\right)\equiv 0\;\left(\mathrm{mod}\;x^{m}-1\right)$.*

**Proof.**  Assume that *C* is generated by $\left(g\left(x\right),g^{\prime }\left(x\right),g^{\prime \prime }\left(x\right)\right)$. Then there exist a(x), b(x) and c(x) such that

$$\begin{array}{ccc} a\left(x\right)\left(g\left(x\right),g^{\prime }\left(x\right),g^{\prime \prime }\left(x\right)\right) & \equiv & \left(g_{11}\left(x\right),g_{12}\left(x\right),g_{13}\left(x\right)\right)\;\left(\mathrm{mod}\;x^{m}-1\right),\\ b\left(x\right)\left(g\left(x\right),g^{\prime }\left(x\right),g^{\prime \prime }\left(x\right)\right) & \equiv & \left(0,g_{22}\left(x\right),g_{23}\left(x\right)\right)\;\left(\mathrm{mod}\;x^{m}-1\right),\\ c\left(x\right)\left(g\left(x\right),g^{\prime }\left(x\right),g^{\prime \prime }\left(x\right)\right) & \equiv & \left(0,0,g_{33}\left(x\right)\right)\;\left(\mathrm{mod}\;x^{m}-1\right). \end{array}$$

Then

$$b\left(x\right)g_{11}\left(x\right)\equiv b\left(x\right)\cdot a\left(x\right)g\left(x\right)\equiv b\left(x\right)g\left(x\right)\cdot a\left(x\right)\equiv 0\cdot a\left(x\right)\equiv 0\;\left(\mathrm{mod}\;x^{m}-1\right).$$

Therefore,

$$g_{11}\left(x\right)g_{22}\left(x\right)\equiv g_{11}\left(x\right)\cdot b\left(x\right)g^{\prime }\left(x\right)\equiv b\left(x\right)g_{11}\left(x\right)\cdot g^{\prime }\left(x\right)\equiv 0\cdot g^{\prime }\left(x\right)\equiv 0\;\left(\mathrm{mod}\;x^{m}-1\right).$$

Further,

$$c\left(x\right)g_{22}\left(x\right)\equiv c\left(x\right)\cdot b\left(x\right)g^{\prime }\left(x\right)\equiv b\left(x\right)\cdot c\left(x\right)g^{\prime }\left(x\right)\equiv b\left(x\right)\cdot 0\equiv 0\;\left(\mathrm{mod}\;x^{m}-1\right).$$

Hence,

$$g_{22}\left(x\right)g_{33}\left(x\right)\equiv g_{22}\left(x\right)\cdot c\left(x\right)g^{\prime \prime }\left(x\right)\equiv c\left(x\right)g_{22}\left(x\right)\cdot g^{\prime \prime }\left(x\right)\equiv 0\cdot g^{\prime \prime }\left(x\right)\equiv 0\;\left(\mathrm{mod}\;x^{m}-1\right).$$

W.L.G. we can assume that $g\left(x\right)\equiv g_{11}\left(x\right)\;\left(\mathrm{mod}\;x^{m}-1\right)$. Then

$$g_{11}\left(x\right)g_{33}\left(x\right)\equiv g\left(x\right)\cdot c\left(x\right)g^{\prime \prime }\left(x\right)\equiv c\left(x\right)g\left(x\right)\cdot g^{\prime \prime }\left(x\right)\equiv 0\cdot g^{\prime \prime }\left(x\right)\equiv 0\;\left(\mathrm{mod}\;x^{m}-1\right).$$

Conversely, suppose that $g_{11}\left(x\right)g_{22}\left(x\right)\equiv g_{11}\left(x\right)g_{33}\left(x\right)\equiv g_{22}\left(x\right)g_{33}\left(x\right)\equiv 0\;\left(\mathrm{mod}\;x^{m}-1\right)$. Consider QC code $C^{\prime }$ of length 2m and index 2, generated by elements $\left(g_{11}\left(x\right),g_{12}\left(x\right)\right)$ and $\left(0,g_{22}\left(x\right)\right)$. Since $g_{11}\left(x\right)g_{22}\left(x\right)\equiv 0\;\left(\mathrm{mod}\;x^{m}-1\right)$, by [13] we get that $C^{\prime }$ can be generated by one element $\left(g_{11}\left(x\right),g_{12}^{\prime }\left(x\right)\right)$. Therefore, *C* is generated by three elements of the form $\left(g_{11}\left(x\right),g_{12}^{\prime }\left(x\right),g_{13}^{\prime }\left(x\right)\right)$, $\left(0,0,g_{23}^{\prime }\left(x\right)\right)$ and $\left(0,0,g_{33}\left(x\right)\right)$. By equality (3) we have that $g_{33}\left(x\right)|g_{23}^{\prime }\left(x\right)$, so *C* is generated by two elements $\left(g_{11}\left(x\right),g_{12}^{\prime }\left(x\right),g_{13}^{\prime }\left(x\right)\right)$ and $g_{3}=\left(0,0,g_{33}\left(x\right)\right)$.

Consider

$$A=\frac{x^{m}-1}{g_{11}\left(x\right)}\cdot \left(g_{11}\left(x\right),g_{12}^{\prime }\left(x\right),g_{13}^{\prime }\left(x\right)\right)=\left(0,\frac{x^{m}-1}{g_{11}\left(x\right)}g_{12}^{\prime }\left(x\right),\frac{x^{m}-1}{g_{11}\left(x\right)}g_{13}^{\prime }\left(x\right)\right).$$

Since $\left(0,g_{22}\left(x\right),g_{23}\left(x\right)\right)\in C$, by equality (2) we have $\frac{x^{m}-1}{g_{11}\left(x\right)}g_{12}^{\prime }\left(x\right)=g_{22}\left(x\right)h$, where h∈R. Since $\left(0,g_{22}\left(x\right)\right)\in C^{\prime }=\langle \left(g_{11}\left(x\right),g_{12}^{\prime }\left(x\right)\right)\rangle$, we have $h\in R^{*}$. Then

$$h^{-1}A=\left(0,g_{22}\left(x\right),\frac{x^{m}-1}{g_{11}\left(x\right)}g_{13}^{\prime }\left(x\right)h^{-1}\right)\in C.$$

Consider QC code $C^{\prime \prime }$ of length 2m and index 2, generated by elements $\left(g_{22}\left(x\right),\frac{x^{m}-1}{g_{11}\left(x\right)}g_{13}^{\prime }\left(x\right)h^{-1}\right)$ and $\left(0,g_{33}\left(x\right)\right)$. Since $g_{22}\left(x\right)g_{33}\left(x\right)\equiv 0\;\left(\mathrm{mod}\;x^{m}-1\right)$, by [13] we get that $C^{\prime \prime }$ can be generated by one element $\left(g_{22}\left(x\right),\frac{x^{m}-1}{g_{11}\left(x\right)}g_{13}^{\prime }\left(x\right)h^{-1}+\alpha g_{33}\left(x\right)h^{-1}\right)=h^{-1}\left(\frac{x^{m}-1}{g_{11}\left(x\right)}g_{12}^{\prime }\left(x\right),\frac{x^{m}-1}{g_{11}\left(x\right)}g_{13}^{\prime }\left(x\right)+\alpha g_{33}\left(x\right)\right)$ for some α∈R. Therefore, *C* is generated by one element

$$\left(g_{11}\left(x\right),g_{12}^{\prime }\left(x\right),g_{13}^{\prime }\left(x\right)+\alpha \frac{g_{11}\left(x\right)g_{33}\left(x\right)}{x^{m}-1}\right),$$

which completes the proof (note that $g_{11}\left(x\right)g_{33}\left(x\right)\equiv 0\;\left(\mathrm{mod}\;x^{m}-1\right)$). □

**Example** **1.** Let m=10, q=3. Then $x^{m}-1=p_{0}p_{1}p_{2}p_{3}$, where $p_{0}=x-1$, $p_{1}=x+1$, $p_{2}=x^{4}+x^{3}+x^{2}+x+1$, $p_{3}=x^{4}+2x^{3}+x^{2}+2x+1$. Let C be a code generated by three elements $\left(p_{0}p_{1}p_{2},0,p_{0}p_{1}\left(-x^{3}+x^{2}+x\right)\right)$, $\left(0,p_{2}p_{3},p_{3}\right)$ and $\left(0,0,p_{0}p_{1}p_{3}\right)$. Then they satisfy Conditions (*) and by Theorem 3 it is a one-generator code. Indeed, it is generated by $\left(p_{0}p_{1}p_{2},xp_{2}p_{3},x^{2}\right)$.

In fact, one part of Theorem 3 is valid without restrictions on the characteristic of the ground field. The proof of Theorem 3 suggests the following:

**Proposition** **4.** *Let C be a QC code of length 3m and index 3, generated by elements $g_{1}=\left(g_{11}\left(x\right),g_{12}\left(x\right),g_{13}\left(x\right)\right)$, $g_{2}=\left(0,g_{22}\left(x\right),g_{23}\left(x\right)\right)$ and $g_{3}=\left(0,0,g_{33}\left(x\right)\right)$, satisfying Conditions (∗). If C is generated by one element then $g_{11}\left(x\right)g_{22}\left(x\right)\equiv g_{11}\left(x\right)g_{33}\left(x\right)\equiv g_{22}\left(x\right)g_{33}\left(x\right)\equiv 0\;\left(\mathrm{mod}\;x^{m}-1\right)$.*

**Theorem** **5.** *Let gcd(q,m)=1. Let C be a QC code of length 3m and index 3, generated by elements $g_{1}=\left(g_{11}\left(x\right),g_{12}\left(x\right),g_{13}\left(x\right)\right)$, $g_{2}=\left(0,g_{22}\left(x\right),g_{23}\left(x\right)\right)$ and $g_{3}=\left(0,0,g_{33}\left(x\right)\right)$, satisfying Conditions (∗). If C is generated by one element then $\gcd\left(g_{11}\left(x\right),g_{22}\left(x\right),g_{33}\left(x\right)\right)|g_{13}\left(x\right)$.*

**Proof.**  As gcd(q,m)=1, we can write

$$g_{12}\left(x\right)=a\left(x\right)\cdot \gcd\left(g_{11}\left(x\right),g_{22}\left(x\right)\right),$$

$$g_{23}\left(x\right)=b\left(x\right)\cdot \gcd\left(g_{22}\left(x\right),g_{33}\left(x\right)\right).$$

As *C* is generated by one element, we have

$$g_{11}\left(x\right)g_{22}\left(x\right)\equiv g_{11}\left(x\right)g_{33}\left(x\right)\equiv g_{22}\left(x\right)g_{33}\left(x\right)\equiv 0\;\left(\mathrm{mod}\;x^{m}-1\right).$$

Hence,

$$g_{11}\left(x\right)g_{22}\left(x\right)=\left(x^{m}-1\right)\gcd\left(g_{11}\left(x\right),g_{22}\left(x\right)\right).$$

From (*) we obtain the following:

$$\begin{array}{ccc} g_{11}\left(x\right)g_{22}\left(x\right)g_{33}\left(x\right) & | & \left(x^{m}-1\right)\left(g_{12}\left(x\right)g_{23}\left(x\right)-g_{13}\left(x\right)g_{22}\left(x\right)\right)\\ \left(x^{m}-1\right)\gcd\left(g_{11}\left(x\right),g_{22}\left(x\right)\right)\;g_{33}\left(x\right) & | & \left(x^{m}-1\right)\left(a\left(x\right)\gcd\left(g_{11},g_{22}\right)\;b\left(x\right)\gcd\left(g_{22},g_{33}\right)-g_{13}g_{22}\right)\\ g_{33}\left(x\right) & | & a\left(x\right)b\left(x\right)\;\gcd\left(g_{22}\left(x\right),g_{33}\left(x\right)\right)-g_{13}\left(x\right)\frac{g_{22}\left(x\right)}{\gcd(g_{11}\left(x\right),g_{22}\left(x\right))} \end{array}$$

Therefore,

$$a\left(x\right)b\left(x\right)\;\gcd\left(g_{22}\left(x\right),g_{33}\left(x\right)\right)-g_{13}\left(x\right)\frac{g_{22}\left(x\right)}{\gcd(g_{11}\left(x\right),g_{22}\left(x\right))}=c\left(x\right)g_{33}\left(x\right)$$

(4) $$g_{13}\left(x\right)\frac{g_{22}\left(x\right)}{\gcd(g_{11}\left(x\right),g_{22}\left(x\right))}=a\left(x\right)b\left(x\right)\;\gcd\left(g_{22}\left(x\right),g_{33}\left(x\right)\right)-c\left(x\right)g_{33}\left(x\right)$$

Note that

(5) $$\begin{array}{ccc} \frac{g_{22}\left(x\right)}{\gcd(g_{11}\left(x\right),g_{22}\left(x\right))} & = & \frac{x^{m}-1}{g_{11}\left(x\right)}, \end{array}$$

(6) $$\begin{array}{ccc} \gcd(g_{22}\left(x\right),g_{33}\left(x\right)) & = & \frac{x^{m}-1}{g_{11}\left(x\right)}\gcd\left(g_{11}\left(x\right),g_{22}\left(x\right),g_{33}\left(x\right)\right), \end{array}$$

(7) $$\begin{array}{ccc} g_{33}\left(x\right) & = & \frac{x^{m}-1}{g_{11}\left(x\right)}\gcd\left(g_{11}\left(x\right),g_{33}\left(x\right)\right). \end{array}$$

Now, divide both sides in (4) by (5). Applying (6) and (7), we have

$$g_{13}\left(x\right)=a\left(x\right)b\left(x\right)\gcd\left(g_{11}\left(x\right),g_{22}\left(x\right),g_{33}\left(x\right)\right)-c\left(x\right)\gcd\left(g_{11}\left(x\right),g_{33}\left(x\right)\right).$$

As $\gcd\left(g_{11}\left(x\right),g_{22}\left(x\right),g_{33}\left(x\right)\right)|\gcd\left(g_{11}\left(x\right),g_{33}\left(x\right)\right)$, for some polynomial d(x) we have

$$g_{13}\left(x\right)=d\left(x\right)\;\gcd\left(g_{11}\left(x\right),g_{22}\left(x\right),g_{33}\left(x\right)\right)$$

Hence,

$$\gcd\left(g_{11}\left(x\right),g_{22}\left(x\right),g_{33}\left(x\right)\right)|g_{13}\left(x\right),$$

which completes the proof. □

## 4. Euclidean Duals of Quasi-Cyclic Codes

Let $f\left(x\right)=a_{0}+a_{1}x+a_{2}x+\cdots +a_{k}x^{k}$ be a polynomial of degree *k*. Then the *reciprocal polynomial* of a(x) is the polynomial

$$f{\left(x\right)}^{*}=x^{\deg f}f\left(x^{-1}\right)=a_{k}+a_{k-1}x+\cdots +a_{0}x^{k}.$$

Let $f\left(x\right)=a_{0}+a_{1}x+\cdots +a_{m-1}x^{m-1}+a_{m}x^{m}$, where *m* is as before, i.e., $R=F\left[x\right]/\langle x^{m}-1\rangle$. We define the *transpose polynomial* $f{\left(x\right)}^{\circ }$ of f(x) as

$$f{\left(x\right)}^{\circ }=x^{m}f\left(x^{-1}\right)=a_{m}+a_{m-1}x+\cdots +a_{2}x^{m-2}+a_{1}x^{m-1}+a_{0}x^{m}.$$

**Lemma** **6.** *(1) If degf(x)≤m then*

$$f{\left(x\right)}^{\circ }=x^{m-\deg f}f{\left(x\right)}^{*},$$

$$f{\left(x\right)}^{\circ }\equiv \left(a_{0}+a_{m}\right)+a_{m-1}x+\ldots +a_{2}x^{m-2}+a_{1}x^{m-1}\;\left(\mathrm{mod}\;x^{m}-1\right).$$

*(2) If degf(x)h(x)≤m then*

$$x^{m}{\left(f(x)h(x)\right)}^{\circ }=f{\left(x\right)}^{\circ }h{\left(x\right)}^{\circ },$$

$${\left(f(x)h(x)\right)}^{\circ }\equiv f{\left(x\right)}^{\circ }h{\left(x\right)}^{\circ }\;\left(\mathrm{mod}\;x^{m}-1\right).$$

**Proof.**  We have

$$f{\left(x\right)}^{\circ }=x^{m}f\left(x^{-1}\right)=x^{m-\deg f}x^{\deg f}f\left(x^{-1}\right)=x^{m-\deg f}f{\left(x\right)}^{*},$$

$$x^{m}{\left(f(x)h(x)\right)}^{\circ }=x^{m}x^{m}f\left(x^{-1}\right)h\left(x^{-1}\right)=f{\left(x\right)}^{\circ }h{\left(x\right)}^{\circ },$$

which proves the lemma. □

We recall the standard inner product on the space $R=F\left[x\right]/\langle x^{m}-1\rangle$. Let $a\left(x\right)=a_{0}+a_{1}x+\cdots +a_{m-1}x^{m-1}$ and $b\left(x\right)=b_{0}+b_{1}x+\cdots +b_{m-1}x^{m-1}$. Then the Euclidean inner product on *R* is defined as

(8) $${\langle a(x),b(x)\rangle }_{e}=a_{0}b_{0}+a_{1}b_{1}+\cdots +a_{m-1}b_{m-1}.$$

It is consistent with the standard dot product between vectors $\left(a_{0},a_{1},\ldots ,a_{m-1}\right)$ and $\left(b_{0},b_{1},\ldots ,b_{m-1}\right)$.

**Lemma** **7** ([10])**.** *Let a(x), b(x), c(x) be polynomials in R. Then*

$${\langle c(x)a(x),b(x)\rangle }_{e}={\langle a\left(x\right),c{\left(x\right)}^{\circ }b\left(x\right)\rangle }_{e}.$$

**Proof.**  It is sufficient to prove the statement for $c\left(x\right)=x^{k}$. We have to show that

$${\langle x^{k}a\left(x\right),b\left(x\right)\rangle }_{e}={\langle a\left(x\right),x^{m-k}b\left(x\right)\rangle }_{e},$$

which is equivalent to the equality

$$\sum_{i=0}^{m-1}a_{i-k}b_{i}=\sum_{i=0}^{m-1}a_{i}b_{i+k},$$

where indices are considered modulo *m*. □

**Remark** **2.** *Lemma 7 explains why $c{\left(x\right)}^{\circ }$ is called the transpose polynomial for c(x). Recall that if φ is an endomorphism of a vector space R, then the adjoint $\varphi^{\circ }$ of φ is defined by the equation ${\langle \varphi (a),b\rangle }_{e}={\langle a,\varphi^{\circ }\left(b\right)\rangle }_{e}$. For symmetric inner products, in the matrix presentation, the adjoint of a matrix is the transpose of the matrix. If we consider the multiplication by c(x) as an endomorphism of R, then its adjoint is the multiplication by $c{\left(x\right)}^{\circ }$.*

**Lemma** **8.** *For f(x)∈F[x], the following statements hold:*

*(1) If f(x) divides $x^{m}-1$, then $f{\left(x\right)}^{\circ }$ divides $x^{m}\left(1-x^{m}\right)$.*

*(2) If f(x) divides $x^{m}-1$, then*

$${\left(\frac{x^{m}-1}{f(x)}\right)}^{\circ }=\frac{x^{m}\left(1-x^{m}\right)}{f{\left(x\right)}^{\circ }}.$$

*(3) If $\deg g_{12}\left(x\right)<\deg g_{22}\left(x\right)$ and $g_{11}\left(x\right)g_{22}\left(x\right)$ divides $\left(x^{m}-1\right)g_{12}\left(x\right)$, then*

$${\left(\frac{\left(x^{m}-1\right)g_{12}\left(x\right)}{g_{11}\left(x\right)g_{22}\left(x\right)}\right)}^{\circ }=\frac{x^{m}\left(1-x^{m}\right)g_{12}{\left(x\right)}^{\circ }}{g_{11}{\left(x\right)}^{\circ }g_{22}{\left(x\right)}^{\circ }}.$$

*(4) If $\deg g_{12}\left(x\right)<\deg g_{22}\left(x\right)$, $\deg g_{23}\left(x\right)<\deg g_{33}\left(x\right)$, $\deg g_{13}\left(x\right)<\deg g\left(x\right)$ and $g_{11}\left(x\right)g_{22}\left(x\right)g_{33}\left(x\right)$ divides $\left(x^{m}-1\right)\left(g_{12}\left(x\right)g_{23}\left(x\right)-g_{13}\left(x\right)g_{22}\left(x\right)\right)$, then*

$${\left(\frac{\left(x^{m}-1\right)\left(g_{12}\left(x\right)g_{23}\left(x\right)-g_{13}\left(x\right)g_{22}\left(x\right)\right)}{g_{11}\left(x\right)g_{22}\left(x\right)g_{33}\left(x\right)}\right)}^{\circ }=\frac{x^{m}\left(1-x^{m}\right)\left(g_{12}{\left(x\right)}^{\circ }g_{23}{\left(x\right)}^{\circ }-g_{13}{\left(x\right)}^{\circ }g_{22}{\left(x\right)}^{\circ }\right)}{g_{11}{\left(x\right)}^{\circ }g_{22}{\left(x\right)}^{\circ }g_{33}{\left(x\right)}^{\circ }}.$$

**Proof.**  We have

$${\left(\frac{x^{m}-1}{f(x)}\right)}^{\circ }=x^{m}\frac{{\left(1/x\right)}^{m}-1}{f(x^{-1})}=x^{m}\frac{1-x^{m}}{x^{m}f\left(x^{-1}\right)}=\frac{x^{m}\left(1-x^{m}\right)}{f{\left(x\right)}^{\circ }}.$$

Let $h\left(x\right)\cdot g_{11}\left(x\right)g_{22}\left(x\right)=\left(x^{m}-1\right)g_{12}\left(x\right)$. Then degh(x)<m and

$$x^{m}h\left(1/x\right)\cdot x^{m}g_{11}\left(1/x\right)x^{m}g_{22}\left(1/x\right)=x^{m}x^{m}\left({\left(1/x\right)}^{m}-1\right)x^{m}g_{12}\left(1/x\right),\;h{\left(x\right)}^{\circ }\cdot g_{11}{\left(x\right)}^{\circ }g_{22}{\left(x\right)}^{\circ }=x^{m}\left(1-x^{m}\right)g_{12}{\left(x\right)}^{\circ }.$$

Let $r\left(x\right)\cdot g_{11}\left(x\right)g_{22}\left(x\right)g_{33}\left(x\right)=\left(x^{m}-1\right)\left(g_{12}\left(x\right)g_{23}\left(x\right)-g_{13}\left(x\right)g_{22}\left(x\right)\right)$. Then degr(x)<m and

$$\begin{array}{c} x^{m}r\left(1/x\right)\cdot x^{m}g_{11}\left(1/x\right)x^{m}g_{22}\left(1/x\right)x^{m}g_{22}\left(1/x\right)=\\ x^{m}x^{m}\left({\left(1/x\right)}^{m}-1\right)x^{m}\left(g_{12}\left(1/x\right)g_{23}\left(1/x\right)-g_{13}\left(1/x\right)g_{22}\left(1/x\right)\right),\\ r{\left(x\right)}^{\circ }\cdot g_{11}{\left(x\right)}^{\circ }g_{22}{\left(x\right)}^{\circ }g_{33}{\left(x\right)}^{\circ }=x^{m}\left(1-x^{m}\right)\left(g_{12}{\left(x\right)}^{\circ }g_{23}{\left(x\right)}^{\circ }-g_{13}{\left(x\right)}^{\circ }g_{22}{\left(x\right)}^{\circ }\right). \end{array}$$

Thus the lemma follows. □

**Remark** **3.** If $f\left(x\right)|\left(x^{m}-1\right)$, then $f{\left(x\right)}^{*}|\left(x^{m}-1\right)$. However, $f{\left(x\right)}^{\circ }$ might not divide $x^{m}-1$, but it divides $x^{m}\left(x^{m}-1\right)$. For example, let m=7, q=2. Then $x^{7}-1=\left(x+1\right)\left(x^{3}+x^{2}+1\right)\left(x^{3}+x+1\right)$. Let $f\left(x\right)=x^{3}+x+1$. Then $f\left(x\right)|\left(x^{m}-1\right)$, $f{\left(x\right)}^{\circ }=x^{7}+x^{6}+x^{4}=x^{4}\left(x^{3}+x^{2}+1\right)∤\;\left(x^{m}-1\right)$, but $f{\left(x\right)}^{\circ }|x^{m}\left(x^{m}-1\right)$.

The inner product (8) can be naturally extended to QC codes of length n=3m and index 3:

$${\langle \left(a(x),b(x),c(x)\right),\left(a^{\prime }\left(x\right),b^{\prime }\left(x\right),c^{\prime }\left(x\right)\right)\rangle }_{e}={\langle a\left(x\right),a^{\prime }\left(x\right)\rangle }_{e}+{\langle b\left(x\right),b^{\prime }\left(x\right)\rangle }_{e}+{\langle c\left(x\right),c^{\prime }\left(x\right)\rangle }_{e}.$$

If *C* is a code in $F^{n}$, then its Euclidean dual code is

$$C^{{⊥}_{e}}=\left\{u\in F^{n}|\langle u,v\rangle =0,\;\mathrm{for}\;\mathrm{all}\;v\in C\right\}.$$

The code *C* is called Euclidean self-orthogonal if $C\subseteq C^{{⊥}_{e}}$, and Euclidean dual-containing if $C⊇C^{{⊥}_{e}}$.

**Theorem** **9.** *Let C be a QC code of length 3m and index 3, generated by elements $g_{1}=\left(g_{11}\left(x\right),g_{12}\left(x\right),g_{13}\left(x\right)\right)$, $g_{2}=\left(0,g_{22}\left(x\right),g_{23}\left(x\right)\right)$ and $g_{3}=\left(0,0,g_{33}\left(x\right)\right)$, satisfying Conditions (∗). Then its Euclidean dual code $C^{{⊥}_{e}}$ is generated by three elements*

$$f_{1}=\left(\frac{x^{m}\left(x^{m}-1\right)}{g_{11}{\left(x\right)}^{\circ }},0,0\right),$$

$$f_{2}=\left(-\frac{x^{m}\left(x^{m}-1\right)g_{12}{\left(x\right)}^{\circ }}{g_{11}{\left(x\right)}^{\circ }g_{22}{\left(x\right)}^{\circ }},\frac{x^{m}\left(x^{m}-1\right)}{g_{22}{\left(x\right)}^{\circ }},0\right),$$

$$f_{3}=\left(\frac{x^{m}\left(x^{m}-1\right)\left(g_{12}{\left(x\right)}^{\circ }g_{23}{\left(x\right)}^{\circ }-g_{13}{\left(x\right)}^{\circ }g_{22}{\left(x\right)}^{\circ }\right)}{g_{11}{\left(x\right)}^{\circ }g_{22}{\left(x\right)}^{\circ }g_{33}{\left(x\right)}^{\circ }},-\frac{x^{m}\left(x^{m}-1\right)g_{23}{\left(x\right)}^{\circ }}{g_{22}{\left(x\right)}^{\circ }g_{33}{\left(x\right)}^{\circ }},\frac{x^{m}\left(x^{m}-1\right)}{g_{33}{\left(x\right)}^{\circ }}\right).$$

*Equivalently, Euclidean dual code $C^{{⊥}_{e}}$ is generated by three elements*

$$h_{3}=\left(h_{33},0,0\right)=\left(\frac{x^{m}-1}{g_{11}{\left(x\right)}^{*}},0,0\right),\;h_{2}=\left(h_{23},h_{22},0\right)=\left(-\frac{x^{m+\deg g_{11}-\deg g_{12}}\left(x^{m}-1\right)g_{12}{\left(x\right)}^{*}}{g_{11}{\left(x\right)}^{*}g_{22}{\left(x\right)}^{*}},\frac{x^{m}-1}{g_{22}{\left(x\right)}^{*}},0\right),$$

$$\begin{array}{c} h_{1}=\left(h_{13},h_{12},h_{11}\right)=\\ \;\left(\frac{\left(x^{m}-1\right)\left(x^{m+\deg g_{11}-\deg g_{12}+\deg g_{22}-\deg g_{23}}g_{12}{\left(x\right)}^{*}g_{23}{\left(x\right)}^{*}-x^{m+\deg g_{11}-\deg g_{13}}g_{13}{\left(x\right)}^{*}g_{22}{\left(x\right)}^{*}\right)}{g_{11}{\left(x\right)}^{*}g_{22}{\left(x\right)}^{*}g_{33}{\left(x\right)}^{*}}\right.,\\ \;\;\;\;\left.-\frac{\left(x^{m}-1\right)x^{m+\deg g_{22}-\deg g_{23}}g_{23}{\left(x\right)}^{*}}{g_{22}{\left(x\right)}^{*}g_{33}{\left(x\right)}^{*}},\frac{x^{m}-1}{g_{33}{\left(x\right)}^{*}}\right). \end{array}$$

**Proof.**  First of all, we show that two generator sets generate the same code:

$$\left(\frac{x^{m}\left(x^{m}-1\right)}{g_{11}{\left(x\right)}^{\circ }},0,0\right)=\left(\frac{x^{m}-1}{g_{11}{\left(x\right)}^{*}},0,0\right)x^{\deg g_{11}},$$

$$\begin{array}{c} \left(-\frac{x^{m}\left(x^{m}-1\right)g_{12}{\left(x\right)}^{\circ }}{g_{11}{\left(x\right)}^{\circ }g_{22}{\left(x\right)}^{\circ }},\frac{x^{m}\left(x^{m}-1\right)}{g_{22}{\left(x\right)}^{\circ }},0\right)\\ \;\equiv \left(-\frac{x^{m}\left(x^{m}-1\right)g_{12}{\left(x\right)}^{\circ }}{g_{11}{\left(x\right)}^{\circ }g_{22}{\left(x\right)}^{\circ }}x^{m},\frac{x^{m}\left(x^{m}-1\right)}{g_{22}{\left(x\right)}^{\circ }},0\right)\;\left(\mathrm{mod}\;x^{m}-1\right)\\ \;\equiv \left(-\frac{x^{m+\deg g_{11}-\deg g_{12}}\left(x^{m}-1\right)g_{12}{\left(x\right)}^{*}}{g_{11}{\left(x\right)}^{*}g_{22}{\left(x\right)}^{*}},\frac{x^{m}-1}{g_{22}{\left(x\right)}^{*}},0\right)x^{\deg g_{22}}\;\left(\mathrm{mod}\;x^{m}-1\right). \end{array}$$

$$\begin{array}{c} \left(\frac{x^{m}\left(x^{m}-1\right)\left(g_{12}{\left(x\right)}^{\circ }g_{23}{\left(x\right)}^{\circ }-g_{13}{\left(x\right)}^{\circ }g_{22}{\left(x\right)}^{\circ }\right)}{g_{11}{\left(x\right)}^{\circ }g_{22}{\left(x\right)}^{\circ }g_{33}{\left(x\right)}^{\circ }},-\frac{x^{m}\left(x^{m}-1\right)g_{23}{\left(x\right)}^{\circ }}{g_{22}{\left(x\right)}^{\circ }g_{33}{\left(x\right)}^{\circ }},\frac{x^{m}\left(x^{m}-1\right)}{g_{33}{\left(x\right)}^{\circ }}\right)\\ \;\;\;\equiv \left(\frac{x^{m}\left(x^{m}-1\right)\left(g_{12}{\left(x\right)}^{\circ }g_{23}{\left(x\right)}^{\circ }-g_{13}{\left(x\right)}^{\circ }g_{22}{\left(x\right)}^{\circ }\right)}{g_{11}{\left(x\right)}^{\circ }g_{22}{\left(x\right)}^{\circ }g_{33}{\left(x\right)}^{\circ }}x^{m}\right.,\\ \;\left.-\frac{x^{m}\left(x^{m}-1\right)g_{23}{\left(x\right)}^{\circ }}{g_{22}{\left(x\right)}^{\circ }g_{33}{\left(x\right)}^{\circ }}x^{m},\frac{x^{m}\left(x^{m}-1\right)}{g_{33}{\left(x\right)}^{\circ }}\right)\;\left(\mathrm{mod}\;x^{m}-1\right)\\ \;\;\;\equiv \left(\frac{\left(x^{m}-1\right)\left(x^{m+\deg g_{11}-\deg g_{12}+\deg g_{22}-\deg g_{23}}g_{12}{\left(x\right)}^{*}g_{23}{\left(x\right)}^{*}-x^{m+\deg g_{11}-\deg g_{13}}g_{13}{\left(x\right)}^{*}g_{22}{\left(x\right)}^{*}\right)}{g_{11}{\left(x\right)}^{*}g_{22}{\left(x\right)}^{*}g_{33}{\left(x\right)}^{*}}\right.,\\ \;\left.-\frac{\left(x^{m}-1\right)x^{m+\deg g_{22}-\deg g_{23}}g_{23}{\left(x\right)}^{*}}{g_{22}{\left(x\right)}^{*}g_{33}{\left(x\right)}^{*}},\frac{x^{m}-1}{g_{33}{\left(x\right)}^{*}}\right)x^{\deg g_{33}}\;\left(\mathrm{mod}\;x^{m}-1\right). \end{array}$$

Let $C^{\prime }$ be a code generated by elements $f_{1}$, $f_{2}$, and $f_{3}$. We show that $C^{\prime }\subseteq C^{{⊥}_{e}}$. We need to show that ${\langle a\left(x\right)g_{i},b\left(x\right)f_{j}\rangle }_{e}=0$ for all a(x),b(x)∈R and i,j=1,2,3. Let us show that ${\langle a\left(x\right)g_{3},b\left(x\right)f_{2}\rangle }_{e}=0$ (other cases are similar). By Lemmas 7 and 8 we have

$$\begin{array}{c} {\langle a\left(x\right)g_{3},b\left(x\right)f_{2}\rangle }_{e}=\\ \;{\langle a\left(x\right)g_{11},b\left(x\right)\left(-\frac{x^{m}\left(x^{m}-1\right)g_{12}{\left(x\right)}^{\circ }}{g_{11}{\left(x\right)}^{\circ }g_{22}{\left(x\right)}^{\circ }}\right)\rangle }_{e}+{\langle a\left(x\right)g_{12}\left(x\right),b\left(x\right)\frac{x^{m}\left(x^{m}-1\right)}{g_{22}{\left(x\right)}^{\circ }},\rangle }_{e}=\\ \;{\langle a\left(x\right)g_{11}\frac{\left(x^{m}-1\right)g_{12}\left(x\right)}{g_{11}\left(x\right)g_{22}\left(x\right)},b\left(x\right)\rangle }_{e}-{\langle a\left(x\right)g_{12}\left(x\right)\frac{x^{m}-1}{g_{22}\left(x\right)},b\left(x\right)\rangle }_{e}=0. \end{array}$$

Thus $C^{\prime }\subseteq C^{{⊥}_{e}}$. We will show now that $\dim C^{\prime }=\dim C^{{⊥}_{e}}$. Note that

$$\dim C^{{⊥}_{e}}=3m-\dim C=\deg g_{11}\left(x\right)+\deg g_{22}\left(x\right)+\deg g_{33}\left(x\right).$$

Generators $h_{1}$, $h_{2}$, $h_{3}$ of the code $C^{\prime }$ satisfy conditions of Theorem 2 (by interchanging coordinate positions 1 and 3 of codewords). Indeed, $h_{11}h_{22}|\left(x^{m}-1\right)h_{12}$, $h_{22}h_{33}|\left(x^{m}-1\right)h_{23}$$\left(x^{m}-1\right)h_{23}$, etc. Therefore, by Theorem 2

$$\dim C^{\prime }=3m-\deg h_{11}\left(x\right)-\deg h_{22}\left(x\right)-\deg h_{33}\left(x\right)=\deg g_{11}\left(x\right)+\deg g_{22}\left(x\right)+\deg g_{33}\left(x\right).$$

Hence, $C^{\prime }=C^{{⊥}_{e}}$, and theorem is proved. □

**Example** **2.** *Let m=13, q=3, Then $x^{m}-1=p_{0}p_{1}p_{2}p_{3}$, where $p_{0}=x-1$, $p_{1}=x^{3}+2x+2$, $p_{2}=x^{3}+x^{2}+2$, $p_{3}=x^{3}+x^{2}+x+2$, $p_{4}=x^{3}+2x^{2}+2x+2$. Let C be a code generated by three elements $\left(p_{0}p_{1},p_{0}\left(x^{2}+x+2\right),p_{0}^{3}\left(x^{2}+x+2\right)\left(x^{4}+x^{3}+2\right)\right)$, $\left(0,p_{0}p_{2}p_{3}p_{4},x^{2}p_{0}p_{2}p_{3}p_{4}\right)$ and $\left(0,0,x^{m}-1\right)$. By Theorem 9 the dual code $C^{{⊥}_{e}}$ is generated by three elements $\left(p_{1}p_{3}p_{4},0,0\right)$, $\left(2x^{3}+x^{2}+x,p_{2},0\right)$ and $\left(2x^{12}+x^{11}+2x^{10}+2x^{8}+1,2x^{11},1\right)$.*

Now, we investigate conditions for *C* to be Euclidean self-orthogonal.

**Theorem** **10.** *Let C be a QC code of length 3m and index 3, generated by elements $g_{1}=\left(g_{11}\left(x\right),g_{12}\left(x\right),g_{13}\left(x\right)\right)$, $g_{2}=\left(0,g_{22}\left(x\right),g_{23}\left(x\right)\right)$ and $g_{3}=\left(0,0,g_{33}\left(x\right)\right)$, satisfying Conditions (∗). Then C is Euclidean self-orthogonal if and only if the following conditions hold:*

*(1) $g_{11}\left(x\right)g_{11}{\left(x\right)}^{\circ }+g_{12}\left(x\right)g_{12}{\left(x\right)}^{\circ }+g_{13}\left(x\right)g_{13}{\left(x\right)}^{\circ }\equiv 0\;\left(\mathrm{mod}\;x^{m}-1\right)$;*

*(2) $g_{12}\left(x\right)g_{22}{\left(x\right)}^{\circ }+g_{13}\left(x\right)g_{23}{\left(x\right)}^{\circ }\equiv 0\;\left(\mathrm{mod}\;x^{m}-1\right)$;*

*(3) $g_{13}\left(x\right)g_{33}{\left(x\right)}^{\circ }\equiv 0\;\left(\mathrm{mod}\;x^{m}-1\right)$;*

*(4) $g_{22}\left(x\right)g_{22}{\left(x\right)}^{\circ }+g_{23}\left(x\right)g_{23}{\left(x\right)}^{\circ }\equiv 0\;\left(\mathrm{mod}\;x^{m}-1\right)$;*

*(5) $g_{23}\left(x\right)g_{33}{\left(x\right)}^{\circ }\equiv 0\;\left(\mathrm{mod}\;x^{m}-1\right)$;*

*(6) $g_{33}\left(x\right)g_{33}{\left(x\right)}^{\circ }\equiv 0\;\left(\mathrm{mod}\;x^{m}-1\right)$.*

**Proof.**  Euclidean self-orthogonality of *C* means that

$${\langle g_{1},b\left(x\right)g_{1}\rangle }_{e}=\langle \left(g_{11}\left(x\right),g_{12}\left(x\right),g_{13}\left(x\right)\right),b\left(x\right){(g_{11}\left(x\right),g_{12}\left(x\right),g_{13}\left(x\right))\rangle }_{e}=0,$$

$${\langle g_{1},b\left(x\right)g_{2}\rangle }_{e}=\langle \left(g_{11}\left(x\right),g_{12}\left(x\right),g_{13}\left(x\right)\right),b\left(x\right){(0,g_{22}\left(x\right),g_{23}\left(x\right))\rangle }_{e}=0,$$

$${\langle g_{1},b\left(x\right)g_{3}\rangle }_{e}=\langle \left(g_{11}\left(x\right),g_{12}\left(x\right),g_{13}\left(x\right)\right),b\left(x\right){(0,0,g_{33}\left(x\right))\rangle }_{e}=0,$$

$${\langle g_{2},b\left(x\right)g_{2}\rangle }_{e}=\langle \left(0,g_{22}\left(x\right),g_{23}\left(x\right)\right),b\left(x\right){(0,g_{22}\left(x\right),g_{23}\left(x\right))\rangle }_{e}=0,$$

$${\langle g_{2},b\left(x\right)g_{3}\rangle }_{e}=\langle \left(0,g_{22}\left(x\right),g_{23}\left(x\right)\right),b\left(x\right){(0,0,g_{33}\left(x\right))\rangle }_{e}=0,$$

$${\langle g_{3},b\left(x\right)g_{3}\rangle }_{e}=\langle \left(0,0,g_{33}\left(x\right)\right),b\left(x\right){(0,0,g_{33}\left(x\right))\rangle }_{e}=0,$$

for any b(x)∈R. This is equivalent to

$${\langle g_{11}\left(x\right)g_{11}{\left(x\right)}^{\circ }+g_{12}\left(x\right)g_{12}{\left(x\right)}^{\circ }+g_{13}\left(x\right)g_{13}{\left(x\right)}^{\circ },b\left(x\right)\rangle }_{e}=0,$$

$${\langle g_{12}\left(x\right)g_{22}{\left(x\right)}^{\circ }+g_{13}\left(x\right)g_{23}{\left(x\right)}^{\circ },b\left(x\right)\rangle }_{e}=0,$$

$${\langle g_{13}\left(x\right)g_{33}{\left(x\right)}^{\circ },b\left(x\right)\rangle }_{e}=0,$$

$${\langle g_{22}\left(x\right)g_{22}{\left(x\right)}^{\circ }+g_{23}\left(x\right)g_{23}{\left(x\right)}^{\circ },b\left(x\right)\rangle }_{e}=0,$$

$${\langle g_{23}\left(x\right)g_{33}{\left(x\right)}^{\circ },b\left(x\right)\rangle }_{e}=0,$$

$${\langle g_{33}\left(x\right)g_{33}{\left(x\right)}^{\circ },b\left(x\right)\rangle }_{e}=0,$$

for any b(x)∈R, which proves the theorem. □

**Example** **3.** *Let C be the code from Example 2. Then C is an Euclidean self-orthogonal [39,12,15]-code (computed by Magma [28]. Its dual is a [39,27,6]-code, which has the same parameters as Best Known Linear Code (BKLC) [29] (but the code in [29] has a complicated structure.*

Similarly, we can give description of Euclidean dual-containing codes.

**Theorem** **11.** *Let C be a QC code of length 3m and index 3, generated by elements $g_{1}=\left(g_{11}\left(x\right),g_{12}\left(x\right),g_{13}\left(x\right)\right)$, $g_{2}=\left(0,g_{22}\left(x\right),g_{23}\left(x\right)\right)$ and $g_{3}=\left(0,0,g_{33}\left(x\right)\right)$, satisfying Conditions (∗). Then C is Euclidean dual-containing if and only if the following conditions hold:*

*(1) $g_{11}\left(x\right)g_{11}{\left(x\right)}^{\circ }$ divides $x^{m}\left(x^{m}-1\right)$;*

*(2) $g_{11}\left(x\right)g_{11}{\left(x\right)}^{\circ }g_{22}\left(x\right)$ divides $x^{m}\left(x^{m}-1\right)g_{12}\left(x\right)$;*

*(3) $g_{11}\left(x\right)g_{11}{\left(x\right)}^{\circ }g_{22}\left(x\right)g_{33}\left(x\right)$ divides $x^{m}\left(x^{m}-1\right)\left(g_{12}\left(x\right)g_{23}\left(x\right)-g_{13}\left(x\right)g_{22}\left(x\right)\right)$;*

*(4) $g_{11}\left(x\right)g_{11}{\left(x\right)}^{\circ }g_{22}\left(x\right)g_{22}{\left(x\right)}^{\circ }$ divides $x^{m}\left(x^{m}-1\right)\left(g_{11}\left(x\right)g_{11}{\left(x\right)}^{\circ }+g_{12}\left(x\right)g_{12}{\left(x\right)}^{\circ }\right)$;*

*(5) $g_{11}\left(x\right)g_{11}{\left(x\right)}^{\circ }g_{22}\left(x\right)g_{22}{\left(x\right)}^{\circ }g_{33}\left(x\right)$ divides $x^{m}\left(x^{m}-1\right)(g_{12}\left(x\right)g_{12}{\left(x\right)}^{\circ }g_{23}\left(x\right)-$*

*$g_{13}\left(x\right)g_{12}{\left(x\right)}^{\circ }g_{22}\left(x\right)-g_{11}\left(x\right)g_{11}{\left(x\right)}^{\circ }g_{23}\left(x\right))$;*

*(6) $g_{11}\left(x\right)g_{11}{\left(x\right)}^{\circ }g_{22}\left(x\right)g_{22}{\left(x\right)}^{\circ }g_{33}\left(x\right)g_{33}{\left(x\right)}^{\circ }$ divides*

$$x^{m}\left(x^{m}-1\right)(g_{12}\left(x\right)g_{12}{\left(x\right)}^{\circ }g_{23}\left(x\right)g_{23}{\left(x\right)}^{\circ }-g_{13}\left(x\right)g_{12}{\left(x\right)}^{\circ }g_{22}\left(x\right)g_{23}{\left(x\right)}^{\circ }-g_{12}\left(x\right)g_{13}{\left(x\right)}^{\circ }g_{22}{\left(x\right)}^{\circ }g_{23}\left(x\right)+\;g_{13}\left(x\right)g_{13}{\left(x\right)}^{\circ }g_{22}\left(x\right)g_{22}{\left(x\right)}^{\circ }+g_{11}\left(x\right)g_{11}{\left(x\right)}^{\circ }g_{23}\left(x\right)g_{23}{\left(x\right)}^{\circ }).$$

**Proof.**  *C* is Euclidean dual-containing if and only if $C^{{⊥}_{e}}$ is Euclidean self-orthogonal. By Theorem 9 it means that

$${\langle \left(\frac{x^{m}\left(x^{m}-1\right)}{g_{11}{\left(x\right)}^{\circ }},0,0\right),\left(\frac{x^{m}\left(x^{m}-1\right)}{g_{11}{\left(x\right)}^{\circ }},0,0\right)b\left(x\right)\rangle }_{e}=0,$$

$${\langle \left(\frac{x^{m}\left(x^{m}-1\right)}{g_{11}{\left(x\right)}^{\circ }},0,0\right),\left(-\frac{x^{m}\left(x^{m}-1\right)g_{12}{\left(x\right)}^{\circ }}{g_{11}{\left(x\right)}^{\circ }g_{22}{\left(x\right)}^{\circ }},\frac{x^{m}\left(x^{m}-1\right)}{g_{22}{\left(x\right)}^{\circ }},0\right)b\left(x\right)\rangle }_{e}=0,$$

$$\begin{array}{c} \langle \left(\frac{x^{m}\left(x^{m}-1\right)}{g_{11}{\left(x\right)}^{\circ }},0,0\right),\\ \;{\left(\frac{x^{m}\left(x^{m}-1\right)\left(g_{12}{\left(x\right)}^{\circ }g_{23}{\left(x\right)}^{\circ }-g_{13}{\left(x\right)}^{\circ }g_{22}{\left(x\right)}^{\circ }\right)}{g_{11}{\left(x\right)}^{\circ }g_{22}{\left(x\right)}^{\circ }g_{33}{\left(x\right)}^{\circ }},-\frac{x^{m}\left(x^{m}-1\right)g_{23}{\left(x\right)}^{\circ }}{g_{22}{\left(x\right)}^{\circ }g_{33}{\left(x\right)}^{\circ }},\frac{x^{m}\left(x^{m}-1\right)}{g_{33}{\left(x\right)}^{\circ }}\right)b\left(x\right)\rangle }_{e}=0, \end{array}$$

$$\;{\langle \left(-\frac{x^{m}\left(x^{m}-1\right)g_{12}{\left(x\right)}^{\circ }}{g_{11}{\left(x\right)}^{\circ }g_{22}{\left(x\right)}^{\circ }},\frac{x^{m}\left(x^{m}-1\right)}{g_{22}{\left(x\right)}^{\circ }},0\right),\left(-\frac{x^{m}\left(x^{m}-1\right)g_{12}{\left(x\right)}^{\circ }}{g_{11}{\left(x\right)}^{\circ }g_{22}{\left(x\right)}^{\circ }},\frac{x^{m}\left(x^{m}-1\right)}{g_{22}{\left(x\right)}^{\circ }},0\right)b\left(x\right)\rangle }_{e}=0,$$

$$\begin{array}{c} \langle \left(-\frac{x^{m}\left(x^{m}-1\right)g_{12}{\left(x\right)}^{\circ }}{g_{11}{\left(x\right)}^{\circ }g_{22}{\left(x\right)}^{\circ }},\frac{x^{m}\left(x^{m}-1\right)}{g_{22}{\left(x\right)}^{\circ }},0\right),\\ \;{\left(\frac{x^{m}\left(x^{m}-1\right)\left(g_{12}{\left(x\right)}^{\circ }g_{23}{\left(x\right)}^{\circ }-g_{13}{\left(x\right)}^{\circ }g_{22}{\left(x\right)}^{\circ }\right)}{g_{11}{\left(x\right)}^{\circ }g_{22}{\left(x\right)}^{\circ }g_{33}{\left(x\right)}^{\circ }},-\frac{x^{m}\left(x^{m}-1\right)g_{23}{\left(x\right)}^{\circ }}{g_{22}{\left(x\right)}^{\circ }g_{33}{\left(x\right)}^{\circ }},\frac{x^{m}\left(x^{m}-1\right)}{g_{33}{\left(x\right)}^{\circ }}\right)b\left(x\right)\rangle }_{e}=0, \end{array}$$

$$\begin{array}{c} \langle \left(\frac{x^{m}\left(x^{m}-1\right)\left(g_{12}{\left(x\right)}^{\circ }g_{23}{\left(x\right)}^{\circ }-g_{13}{\left(x\right)}^{\circ }g_{22}{\left(x\right)}^{\circ }\right)}{g_{11}{\left(x\right)}^{\circ }g_{22}{\left(x\right)}^{\circ }g_{33}{\left(x\right)}^{\circ }},-\frac{x^{m}\left(x^{m}-1\right)g_{23}{\left(x\right)}^{\circ }}{g_{22}{\left(x\right)}^{\circ }g_{33}{\left(x\right)}^{\circ }},\frac{x^{m}\left(x^{m}-1\right)}{g_{33}{\left(x\right)}^{\circ }}\right),\\ \;{\left(\frac{x^{m}\left(x^{m}-1\right)\left(g_{12}{\left(x\right)}^{\circ }g_{23}{\left(x\right)}^{\circ }-g_{13}{\left(x\right)}^{\circ }g_{22}{\left(x\right)}^{\circ }\right)}{g_{11}{\left(x\right)}^{\circ }g_{22}{\left(x\right)}^{\circ }g_{33}{\left(x\right)}^{\circ }},-\frac{x^{m}\left(x^{m}-1\right)g_{23}{\left(x\right)}^{\circ }}{g_{22}{\left(x\right)}^{\circ }g_{33}{\left(x\right)}^{\circ }},\frac{x^{m}\left(x^{m}-1\right)}{g_{33}{\left(x\right)}^{\circ }}\right)b\left(x\right)\rangle }_{e}=0, \end{array}$$

for any b(x)∈R. This is equivalent to

$$\frac{x^{m}\left(x^{m}-1\right)}{g_{11}{\left(x\right)}^{\circ }}{\left(\frac{x^{m}\left(x^{m}-1\right)}{g_{11}{\left(x\right)}^{\circ }}\right)}^{\circ }\equiv 0\;\left(\mathrm{mod}\;x^{m}-1\right),$$

$$\frac{x^{m}\left(x^{m}-1\right)}{g_{11}{\left(x\right)}^{\circ }}{\left(\frac{x^{m}\left(x^{m}-1\right)g_{12}{\left(x\right)}^{\circ }}{g_{11}{\left(x\right)}^{\circ }g_{22}{\left(x\right)}^{\circ }}\right)}^{\circ }\equiv 0\;\left(\mathrm{mod}\;x^{m}-1\right),$$

$$\frac{x^{m}\left(x^{m}-1\right)}{g_{11}{\left(x\right)}^{\circ }}{\left(\frac{x^{m}\left(x^{m}-1\right)\left(g_{12}{\left(x\right)}^{\circ }g_{23}{\left(x\right)}^{\circ }-g_{13}{\left(x\right)}^{\circ }g_{22}{\left(x\right)}^{\circ }\right)}{g_{11}{\left(x\right)}^{\circ }g_{22}{\left(x\right)}^{\circ }g_{33}{\left(x\right)}^{\circ }}\right)}^{\circ }\equiv 0\;\left(\mathrm{mod}\;x^{m}-1\right),$$

$$\frac{x^{m}\left(x^{m}-1\right)g_{12}{\left(x\right)}^{\circ }}{g_{11}{\left(x\right)}^{\circ }g_{22}{\left(x\right)}^{\circ }}{\left(\frac{x^{m}\left(x^{m}-1\right)g_{12}{\left(x\right)}^{\circ }}{g_{11}{\left(x\right)}^{\circ }g_{22}{\left(x\right)}^{\circ }}\right)}^{\circ }+\frac{x^{m}\left(x^{m}-1\right)}{g_{22}{\left(x\right)}^{\circ }}{\left(\frac{x^{m}\left(x^{m}-1\right)}{g_{22}{\left(x\right)}^{\circ }}\right)}^{\circ }\equiv 0\;\left(\mathrm{mod}\;x^{m}-1\right),$$

$$\begin{array}{c} \frac{x^{m}\left(x^{m}-1\right)g_{12}{\left(x\right)}^{\circ }}{g_{11}{\left(x\right)}^{\circ }g_{22}{\left(x\right)}^{\circ }}{\left(\frac{x^{m}\left(x^{m}-1\right)\left(g_{12}{\left(x\right)}^{\circ }g_{23}{\left(x\right)}^{\circ }-g_{13}{\left(x\right)}^{\circ }g_{22}{\left(x\right)}^{\circ }\right)}{g_{11}{\left(x\right)}^{\circ }g_{22}{\left(x\right)}^{\circ }g_{33}{\left(x\right)}^{\circ }}\right)}^{\circ }+\\ \frac{x^{m}\left(x^{m}-1\right)}{g_{22}{\left(x\right)}^{\circ }}{\left(\frac{x^{m}\left(x^{m}-1\right)g_{23}{\left(x\right)}^{\circ }}{g_{22}{\left(x\right)}^{\circ }g_{33}{\left(x\right)}^{\circ }}\right)}^{\circ }\equiv 0\;\left(\mathrm{mod}\;x^{m}-1\right), \end{array}$$

$$\begin{array}{c} \frac{x^{m}\left(x^{m}-1\right)(g_{12}{\left(x\right)}^{\circ }g_{23}{\left(x\right)}^{\circ }-g_{13}{\left(x\right)}^{\circ }g_{22}{\left(x\right)}^{\circ }}{g_{11}{\left(x\right)}^{\circ }g_{22}{\left(x\right)}^{\circ }g_{33}{\left(x\right)}^{\circ }}{\left(\frac{x^{m}\left(x^{m}-1\right)\left(g_{12}{\left(x\right)}^{\circ }g_{23}{\left(x\right)}^{\circ }-g_{13}{\left(x\right)}^{\circ }g_{22}{\left(x\right)}^{\circ }\right)}{g_{11}{\left(x\right)}^{\circ }g_{22}{\left(x\right)}^{\circ }g_{33}{\left(x\right)}^{\circ }}\right)}^{\circ }+\\ \frac{x^{m}\left(x^{m}-1\right)g_{23}{\left(x\right)}^{\circ }}{g_{22}{\left(x\right)}^{\circ }g_{33}{\left(x\right)}^{\circ }}{\left(\frac{x^{m}\left(x^{m}-1\right)g_{23}{\left(x\right)}^{\circ }}{g_{22}{\left(x\right)}^{\circ }g_{33}{\left(x\right)}^{\circ }}\right)}^{\circ }\equiv 0\;\left(\mathrm{mod}\;x^{m}-1\right), \end{array}$$

i.e.,

$$\frac{x^{m}\left(x^{m}-1\right)}{g_{11}{\left(x\right)}^{\circ }}\cdot \frac{\left(x^{m}-1\right)}{g_{11}\left(x\right)}\equiv 0\;\left(\mathrm{mod}\;x^{m}-1\right),$$

$$\frac{x^{m}\left(x^{m}-1\right)}{g_{11}{\left(x\right)}^{\circ }}\cdot \frac{\left(x^{m}-1\right)g_{12}\left(x\right)}{g_{11}\left(x\right)g_{22}\left(x\right)}\equiv 0\;\left(\mathrm{mod}\;x^{m}-1\right),$$

$$\frac{x^{m}\left(x^{m}-1\right)}{g_{11}{\left(x\right)}^{\circ }}\left(\frac{\left(x^{m}-1\right)\left(g_{12}\left(x\right)g_{23}\left(x\right)-g_{13}\left(x\right)g_{22}\left(x\right)\right)}{g_{11}\left(x\right)g_{22}\left(x\right)g_{33}\left(x\right)}\right)\equiv 0\;\left(\mathrm{mod}\;x^{m}-1\right),$$

$$\frac{x^{m}\left(x^{m}-1\right)g_{12}{\left(x\right)}^{\circ }}{g_{11}{\left(x\right)}^{\circ }g_{22}{\left(x\right)}^{\circ }}\cdot \frac{\left(x^{m}-1\right)g_{12}\left(x\right)}{g_{11}\left(x\right)g_{22}\left(x\right)}+\frac{x^{m}\left(x^{m}-1\right)}{g_{22}{\left(x\right)}^{\circ }}\cdot \frac{\left(x^{m}-1\right)}{g_{22}\left(x\right)}\equiv 0\;\left(\mathrm{mod}\;x^{m}-1\right),$$

$$\begin{array}{c} \frac{x^{m}\left(x^{m}-1\right)g_{12}{\left(x\right)}^{\circ }}{g_{11}{\left(x\right)}^{\circ }g_{22}{\left(x\right)}^{\circ }}\left(\frac{\left(x^{m}-1\right)\left(g_{12}\left(x\right)g_{23}\left(x\right)-g_{13}\left(x\right)g_{22}\left(x\right)\right)}{g_{11}\left(x\right)g_{22}\left(x\right)g_{33}\left(x\right)}\right)+\\ \;\;\;\;\frac{x^{m}\left(x^{m}-1\right)}{g_{22}{\left(x\right)}^{\circ }}\left(\frac{\left(x^{m}-1\right)g_{23}\left(x\right)}{g_{22}\left(x\right)g_{33}\left(x\right)}\right)\equiv 0\;\left(\mathrm{mod}\;x^{m}-1\right), \end{array}$$

$$\begin{array}{c} \frac{x^{m}\left(x^{m}-1\right)\left(g_{12}{\left(x\right)}^{\circ }g_{23}{\left(x\right)}^{\circ }-g_{13}{\left(x\right)}^{\circ }g_{22}{\left(x\right)}^{\circ }\right)}{g_{11}{\left(x\right)}^{\circ }g_{22}{\left(x\right)}^{\circ }g_{33}{\left(x\right)}^{\circ }}\left(\frac{\left(x^{m}-1\right)\left(g_{12}\left(x\right)g_{23}\left(x\right)-g_{13}\left(x\right)g_{22}\left(x\right)\right)}{g_{11}\left(x\right)g_{22}\left(x\right)g_{33}\left(x\right)}\right)+\\ \;\;\;\;\frac{x^{m}\left(x^{m}-1\right)g_{23}{\left(x\right)}^{\circ }}{g_{22}{\left(x\right)}^{\circ }g_{33}{\left(x\right)}^{\circ }}\left(\frac{x^{m}\left(x^{m}-1\right)g_{23}\left(x\right)}{g_{22}\left(x\right)g_{33}\left(x\right)}\right)\equiv 0\;\left(\mathrm{mod}\;x^{m}-1\right), \end{array}$$

which proves the theorem. □

In terms of reciprocal polynomials Theorem 10 can be reformulated as follows.

**Corollary** **12.** *Let C be a QC code of length 3m and index 3, generated by elements $g_{1}=\left(g_{11}\left(x\right),g_{12}\left(x\right),g_{13}\left(x\right)\right)$, $g_{2}=\left(0,g_{22}\left(x\right),g_{23}\left(x\right)\right)$ and $g_{3}=\left(0,0,g_{33}\left(x\right)\right)$, satisfying Conditions (∗). Then C is self-orthogonal if and only if the following conditions hold:*

(1) $x^{\deg g_{12}+\deg g_{13}}g_{11}\left(x\right)g_{11}{\left(x\right)}^{*}+x^{\deg g_{11}+\deg g_{13}}g_{12}\left(x\right)g_{12}{\left(x\right)}^{*}+x^{\deg g_{11}+\deg g_{12}}$$g_{13}\left(x\right)g_{13}{\left(x\right)}^{*}\equiv 0\;\left(\mathrm{mod}\;x^{m}-1\right)$;

(2) $x^{\deg g_{23}}g_{12}\left(x\right)g_{22}{\left(x\right)}^{*}+x^{\deg g_{22}}g_{13}\left(x\right)g_{23}{\left(x\right)}^{*}\equiv 0\;\left(\mathrm{mod}\;x^{m}-1\right)$;

(3) $g_{13}\left(x\right)g_{33}{\left(x\right)}^{*}\equiv 0\;\left(\mathrm{mod}\;x^{m}-1\right)$;

(4) $x^{\deg g_{23}}g_{22}\left(x\right)g_{22}{\left(x\right)}^{*}+x^{\deg g_{22}}g_{23}\left(x\right)g_{23}{\left(x\right)}^{*}\equiv 0\;\left(\mathrm{mod}\;x^{m}-1\right)$;

(5) $g_{23}\left(x\right)g_{33}{\left(x\right)}^{*}\equiv 0\;\left(\mathrm{mod}\;x^{m}-1\right)$;

(6) $g_{33}\left(x\right)g_{33}{\left(x\right)}^{*}\equiv 0\;\left(\mathrm{mod}\;x^{m}-1\right)$.

**Example** **4.** *Let m=10, q=3, and let C be a code generated by $\left(g_{11}\left(x\right),g_{12}\left(x\right),g_{13})\left(x\right)\right)$, where $g_{11}\left(x\right)=1$, $g_{12}\left(x\right)=x^{9}+2x^{8}+x^{7}+x^{6}+x^{5}+1$, $g_{13}=2x^{9}+2x^{8}+x^{7}+x^{6}+x^{4}+x^{3}+x^{2}+1$. Then C is an Euclidean self-orthogonal [30,10,12]-code, and its dual is a [30,20,6]-code. The code $C^{{⊥}_{e}}$ is optimal [29].*

**Example** **5.** *Let m=10, q=3. Then $x^{m}-1=p_{0}p_{1}p_{2}p_{3}$, where $p_{0}=x-1$, $p_{1}=x+1$, $p_{2}=x^{4}+x^{3}+x^{2}+x+1$, $p_{3}=x^{4}+2x^{3}+x^{2}+2x+1$. Let C be a code generated by $\left(g_{11}\left(x\right),g_{12}\left(x\right),g_{13})\left(x\right)\right)$, where $g_{11}\left(x\right)=p_{0}p_{1}$, $g_{12}\left(x\right)=p_{0}p_{1}\left(x^{3}+x^{2}+x+2\right)$, $g_{13}=p_{0}^{2}p_{1}\left(x^{5}+2x^{3}+2x^{2}+2\right)$. Then C is an Euclidean self-orthogonal [30,8,15]-code, and its dual is a [30,22,5]-code. Both codes are optimal [29].*

**Example** **6.** *Let m=14, q=3. Then $x^{m}-1=p_{0}p_{1}p_{2}p_{3}$, where $p_{0}=x-1$, $p_{1}=x+1$, $p_{2}=x^{6}+x^{5}+x^{4}+x^{3}+x^{2}+x+1$, $p_{3}=x^{6}+2x^{5}+x^{4}+2x^{3}+x^{2}+2x+1$. Let C be a code generated by $\left(g_{11}\left(x\right),g_{12}\left(x\right),g_{13}\left(x\right)\right)$, where $g_{11}\left(x\right)=p_{0}$, $g_{12}\left(x\right)=p_{0}x\left(x^{11}+2x^{10}+x^{8}+2x^{7}+x^{5}+x^{3}+x+1\right)$, $g_{13}=p_{0}\left(x^{2}+1\right)\left(x^{5}+x^{4}+x^{2}+1\right)$. Then C is an Euclidean self-orthogonal [42,13,18] code with the same parameters as BKLC, and its dual is a [42,29,6] almost BKLC [29].*

## 5. Hermitian Duals of Quasi-Cyclic Codes

In this section we consider codes over the field $F_{q^{2}}$ of $q^{2}$ elements. Let $a\left(x\right)=a_{0}+a_{1}x+\cdots +a_{m-1}x^{m-1}$ and $b\left(x\right)=b_{0}+b_{1}x+\cdots +b_{m-1}x^{m-1}$, where $a_{i}$, $b_{i}\in F_{q^{2}}$ for any 0≤i≤m−1. For $c\in F_{q^{2}}$ we define $\bar{c}=c^{q}$ and

$$\bar{a}\left(x\right)=\bar{a_{0}}+\bar{a_{1}}x+\cdots +\bar{a_{m-1}}x^{m-1}.$$

Then the Hermitian inner product on *R* is defined as

(9) $${\langle a(x),b(x)\rangle }_{h}={\langle a\left(x\right),\bar{b}\left(x\right)\rangle }_{e}=a_{0}\bar{b_{0}}+a_{1}\bar{b_{1}}+\cdots +a_{m-1}\bar{b_{m-1}}.$$

If *C* is a code in ${\left(F_{q^{2}}\right)}^{2m}$, then its Hermitian dual is

$$C^{{⊥}_{h}}=\left\{u\in {\left(F_{q^{2}}\right)}^{2m}|{\langle u,v\rangle }_{h}=0,\;\mathrm{for}\;\mathrm{all}\;v\in C\right\}.$$

The code *C* is called Hermitian self-orthogonal if $C\subseteq C^{{⊥}_{h}}$, and Hermitian dual-containing if $C⊇C^{{⊥}_{h}}$. We investigate Hermitian codes following a similar pattern to that used for Euclidean codes.

Let $f\left(x\right)=a_{0}+a_{1}x+\cdots +a_{m-1}x^{m-1}+a_{m}x^{m}$. We define the *conjugate transpose polynomial* $f{\left(x\right)}^{†}$ of f(x) as

$$f{\left(x\right)}^{†}=x^{m}\bar{f}\left(x^{-1}\right)=\bar{a_{m}}+\bar{a_{m-1}}x+\cdots +\bar{a_{2}}x^{m-2}+\bar{a_{1}}x^{m-1}+\bar{a_{0}}x^{m}.$$

**Lemma** **13.** *If degf(x)≤m, degh(x)≤m and degf(x)h(x)≤m, then*

(1) $f{\left(x\right)}^{†}=x^{m-\deg f}{\bar{f}}^{*}\left(x\right)$;

*(2) $x^{m}{\left(f(x)h(x)\right)}^{†}=f{\left(x\right)}^{†}h{\left(x\right)}^{†}$;*

*(3) $f{\left(x\right)}^{†}\equiv \left(\bar{a_{0}}+\bar{a_{m}}\right)+\bar{a_{m-1}}x+\cdots +\bar{a_{2}}x^{m-2}+\bar{a_{1}}x^{m-1}\;\left(\mathrm{mod}\;x^{m}-1\right)$;*

*(4) ${\left(f(x)h(x)\right)}^{†}\equiv f{\left(x\right)}^{†}h{\left(x\right)}^{†}\;\left(\mathrm{mod}\;x^{m}-1\right)$.*

**Proof.**  It is similar to the proof of Lemma 6. □

The next lemma is an analog of Lemma 7 and it can be proved in the same way.

**Lemma** **14.** *Let a(x), b(x), c(x) be polynomials in R. Then*

$${\langle a(x)c(x),b(x)\rangle }_{h}={\langle a\left(x\right),b\left(x\right)c{\left(x\right)}^{†}\rangle }_{h}.$$

The inner product (9) can be naturally extended to QC codes of length 3m and index 3:

$${\langle \left(a(x),b(x),c(x)\right),\left(a^{\prime }\left(x\right),b^{\prime }\left(x\right),c^{\prime }\left(x\right)\right)\rangle }_{h}={\langle a\left(x\right),a^{\prime }\left(x\right)\rangle }_{h}+\langle b\left(x\right),b^{\prime }\left(x\right)+{\langle c\left(x\right),c^{\prime }\left(x\right)\rangle }_{h}.$$

**Lemma** **15.** *(1) If f(x) divides $x^{m}-1$, then $f{\left(x\right)}^{†}$ divides $x^{m}\left(1-x^{m}\right)$.*

*(2) If f(x) divides $x^{m}-1$, then*

$${\left(\frac{x^{m}-1}{f(x)}\right)}^{†}=\frac{x^{m}\left(1-x^{m}\right)}{f{\left(x\right)}^{†}}.$$

*(3) If $\deg g_{12}\left(x\right)<\deg g_{22}\left(x\right)$ and $g_{11}\left(x\right)g_{22}\left(x\right)$ divides $\left(x^{m}-1\right)g_{12}\left(x\right)$, then*

$${\left(\frac{\left(x^{m}-1\right)g_{12}\left(x\right)}{g_{11}\left(x\right)g_{22}\left(x\right)}\right)}^{†}=\frac{x^{m}\left(1-x^{m}\right)g_{12}{\left(x\right)}^{†}}{g_{11}{\left(x\right)}^{†}g_{22}{\left(x\right)}^{†}}.$$

*(4) If $\deg g_{12}\left(x\right)<\deg g_{22}\left(x\right)$, $\deg g_{23}\left(x\right)<\deg g_{33}\left(x\right)$, $\deg g_{13}\left(x\right)<\deg g\left(x\right)$ and $g_{11}\left(x\right)g_{22}\left(x\right)g_{33}\left(x\right)$ divides $\left(x^{m}-1\right)\left(g_{12}\left(x\right)g_{23}\left(x\right)-g_{13}\left(x\right)g_{22}\left(x\right)\right)$, then*

$${\left(\frac{\left(x^{m}-1\right)\left(g_{12}\left(x\right)g_{23}\left(x\right)-g_{13}\left(x\right)g_{22}\left(x\right)\right)}{g_{11}\left(x\right)g_{22}\left(x\right)g_{33}\left(x\right)}\right)}^{†}=\frac{x^{m}\left(1-x^{m}\right)\left(g_{12}{\left(x\right)}^{†}g_{23}{\left(x\right)}^{†}-g_{13}{\left(x\right)}^{†}g_{22}{\left(x\right)}^{†}\right)}{g_{11}{\left(x\right)}^{†}g_{22}{\left(x\right)}^{†}g_{33}{\left(x\right)}^{†}}.$$

**Proof.**  It is similar to the proof of Lemma 8. □

**Theorem** **16.** *Let C be a QC code of length 3m and index 3, generated by elements $g_{1}=\left(g_{11}\left(x\right),g_{12}\left(x\right),g_{13}\left(x\right)\right)$, $g_{2}=\left(0,g_{22}\left(x\right),g_{23}\left(x\right)\right)$ and $g_{3}=\left(0,0,g_{33}\left(x\right)\right)$, satisfying Conditions (∗). Then its Hermitian dual code $C^{{⊥}_{h}}$ is generated by three elements*

$$\left(\frac{x^{m}\left(x^{m}-1\right)}{g_{11}{\left(x\right)}^{†}},0,0\right),$$

$$\left(-\frac{x^{m}\left(x^{m}-1\right)g_{12}{\left(x\right)}^{†}}{g_{11}{\left(x\right)}^{†}g_{22}{\left(x\right)}^{†}},\frac{x^{m}\left(x^{m}-1\right)}{g_{22}{\left(x\right)}^{†}},0\right),$$

$$\left(\frac{x^{m}\left(x^{m}-1\right)\left(g_{12}{\left(x\right)}^{†}g_{23}{\left(x\right)}^{†}-g_{13}{\left(x\right)}^{†}g_{22}{\left(x\right)}^{†}\right)}{g_{11}{\left(x\right)}^{†}g_{22}{\left(x\right)}^{†}g_{33}{\left(x\right)}^{†}},-\frac{x^{m}\left(x^{m}-1\right)g_{23}{\left(x\right)}^{†}}{g_{22}{\left(x\right)}^{†}g_{33}{\left(x\right)}^{†}},\frac{x^{m}\left(x^{m}-1\right)}{g_{33}{\left(x\right)}^{†}}\right).$$

The next two theorems give characterizations of Hermitian self-orthogonal and Hermitian dual-containing codes.

**Theorem** **17.** *Let C be a QC code of length 3m and index 3, generated by elements $g_{1}=\left(g_{11}\left(x\right),g_{12}\left(x\right),g_{13}\left(x\right)\right)$, $g_{2}=\left(0,g_{22}\left(x\right),g_{23}\left(x\right)\right)$ and $g_{3}=\left(0,0,g_{33}\left(x\right)\right)$, satisfying Conditions (∗). Then C is Hermitian self-orthogonal if and only if the following conditions hold:*

*(1) $g_{11}\left(x\right)g_{11}{\left(x\right)}^{†}+g_{12}\left(x\right)g_{12}{\left(x\right)}^{†}+g_{13}\left(x\right)g_{13}{\left(x\right)}^{†}\equiv 0\;\left(\mathrm{mod}\;x^{m}-1\right)$;*

*(2) $g_{12}\left(x\right)g_{22}{\left(x\right)}^{†}+g_{13}\left(x\right)g_{23}{\left(x\right)}^{†}\equiv 0\;\left(\mathrm{mod}\;x^{m}-1\right)$;*

*(3) $g_{13}\left(x\right)g_{33}{\left(x\right)}^{†}\equiv 0\;\left(\mathrm{mod}\;x^{m}-1\right)$.*

*(4) $g_{22}\left(x\right)g_{22}{\left(x\right)}^{†}+g_{23}\left(x\right)g_{23}{\left(x\right)}^{†}\equiv 0\;\left(\mathrm{mod}\;x^{m}-1\right)$;*

*(5) $g_{23}\left(x\right)g_{33}{\left(x\right)}^{†}\equiv 0\;\left(\mathrm{mod}\;x^{m}-1\right)$;*

*(6) $g_{33}\left(x\right)g_{33}{\left(x\right)}^{†}\equiv 0\;\left(\mathrm{mod}\;x^{m}-1\right)$.*

**Example** **7.** *Let m=7, q=2, $F_{q^{2}}=F_{2}\left[w\right]$. Then $x^{m}-1=p_{0}p_{1}p_{2}$, where $p_{0}=x+1$, $p_{1}=x^{3}+x+1$, $p_{2}=x^{3}+x^{2}+1$. Let C be a code generated by $\left(g_{11}\left(x\right),g_{12}\left(x\right),g_{13})\left(x\right)\right)$, where $g_{11}\left(x\right)=p_{1}$, $g_{12}\left(x\right)=p_{1}\left(x+w\right)$, $g_{13}=p_{0}p_{1}\left(x+w^{2}\right)$. Then C is a Hermitian self-orthogonal [21,4,14]-code, and its dual is a [21,17,3]-code. Both codes are optimal [29].*

**Example** **8.** *Let m=7, q=2, $F_{q^{2}}=F_{2}\left[w\right]$. Let C be a code generated by three elements $\left(1,1,x^{2}+x+w\right)$, $\left(0,p_{0},x^{5}+x^{4}+w^{2}x^{3}+wx^{2}+w\right)$ and $\left(0,0,p_{1}p_{2}\right)$. Then C is a [21,14,5]-code, and its Hermitian dual $C^{⊥}$ is a [21,7,11]-code. Code C is a BKLC and $C^{⊥}$ is optimal [29].*

**Theorem** **18.** *Let C be a QC code of length 3m and index 3, generated by elements $g_{1}=\left(g_{11}\left(x\right),g_{12}\left(x\right),g_{13}\left(x\right)\right)$, $g_{2}=\left(0,g_{22}\left(x\right),g_{23}\left(x\right)\right)$ and $g_{3}=\left(0,0,g_{33}\left(x\right)\right)$, satisfying Conditions (∗). Then C is Hermitian dual-containing if and only if the following conditions hold:*

*(1) $g_{11}\left(x\right)g_{11}{\left(x\right)}^{†}$ divides $x^{m}\left(x^{m}-1\right)$;*

*(2) $g_{11}\left(x\right)g_{11}{\left(x\right)}^{†}g_{22}\left(x\right)$ divides $x^{m}\left(x^{m}-1\right)g_{12}\left(x\right)$;*

*(3) $g_{11}\left(x\right)g_{11}{\left(x\right)}^{†}g_{22}\left(x\right)g_{33}\left(x\right)$ divides $x^{m}\left(x^{m}-1\right)\left(g_{12}\left(x\right)g_{23}\left(x\right)-g_{13}\left(x\right)g_{22}\left(x\right)\right)$.*

*(4) $g_{11}\left(x\right)g_{11}{\left(x\right)}^{†}g_{22}\left(x\right)g_{22}{\left(x\right)}^{†}$ divides $x^{m}\left(x^{m}-1\right)\left(g_{11}\left(x\right)g_{11}{\left(x\right)}^{†}+g_{12}\left(x\right)g_{12}{\left(x\right)}^{†}\right)$.*

*(5) $g_{11}\left(x\right)g_{11}{\left(x\right)}^{†}g_{22}\left(x\right)g_{22}{\left(x\right)}^{†}g_{33}\left(x\right)$ divides $x^{m}\left(x^{m}-1\right)(g_{12}\left(x\right)g_{12}{\left(x\right)}^{†}g_{23}\left(x\right)-$*

*$g_{13}\left(x\right)g_{12}{\left(x\right)}^{†}g_{22}\left(x\right)-g_{11}\left(x\right)g_{11}{\left(x\right)}^{†}g_{23}\left(x\right))$.*

*(6) $g_{11}\left(x\right)g_{11}{\left(x\right)}^{†}g_{22}\left(x\right)g_{22}{\left(x\right)}^{†}g_{33}\left(x\right)g_{33}{\left(x\right)}^{†}$ divides*

$$x^{m}\left(x^{m}-1\right)\left(g_{12}\left(x\right)g_{12}{\left(x\right)}^{†}g_{23}\left(x\right)g_{23}{\left(x\right)}^{†}-g_{13}\left(x\right)g_{12}{\left(x\right)}^{†}g_{22}\left(x\right)g_{23}{\left(x\right)}^{†}-g_{12}\left(x\right)g_{13}{\left(x\right)}^{†}g_{22}{\left(x\right)}^{†}g_{23}\left(x\right)+\;g_{13}\left(x\right)g_{13}{\left(x\right)}^{†}g_{22}\left(x\right)g_{22}{\left(x\right)}^{†}+g_{11}\left(x\right)g_{11}{\left(x\right)}^{†}g_{23}\left(x\right)g_{23}{\left(x\right)}^{†}\right).$$

## 6. Conclusions

In this paper we gave a classification of QC codes of index 3 over finite fields. A QC code of index 3 is generated by at most three elements. We gave a necessary and sufficient condition for a QC code to be generated by one element. We described duals of QC codes with respect to the Euclidean and Hermitian inner products. Moreover, we described self-orthogonal and dual-containing codes with respect to these inner products.

## Data Availability

The original contributions presented in this study are included in the article. Further inquiries can be directed to the corresponding author(s).
